# The Effect of *Lactobacillus crispatus* and *Lactobacillus* rhamnosusCulture Supernatants on Expression of Autophagy Genes and HPV *E6* and *E7* Oncogenes in The HeLa Cell Line 

**DOI:** 10.22074/cellj.2016.3833

**Published:** 2016-01-17

**Authors:** Elahe Motevaseli, Rosa Azam, Seyed Mohammad Akrami, Mohammadali Mazlomy, Mojtaba Saffari, Mohammad Hossein Modarressi, Maryam Daneshvar, Soudeh Ghafouri-Fard

**Affiliations:** 1 Department of Molecular Medicine, School of Advanced Technologies in Medicine, Tehran University of Medical Sciences, Tehran, Iran; 2 Department of Medical Genetics, Tehran University of Medical Sciences, Tehran, Iran; 3 Department of Medical Biotechnology, School of Advanced Technologies in Medicine, Tehran University of Medical Sciences, Tehran, Iran; 4 Department of Medical Genetics, Shahid Beheshti University of Medical Sciences, Tehran, Iran

**Keywords:** HPV, Lactobacillus, Autophagy

## Abstract

**Objective:**

The aim of this study was to clarify the mechanism by which lactobacilli exert their cytotoxic effects on cervical cancer cells. In addition, we aimed to evalu- ate the effect of lactobacilli on the expression of human papilloma virus (HPV) onco- genes.

**Materials and Methods:**

In this experimental study, using quantitative real-time polymer- ase chain reaction (PCR), we analyzed the expression of *CASP3* and three autophagy genes [*ATG14*, *BECN1* and alpha 2 catalytic subunit of AMPK (*PRKAA2*)] along with HPV18 *E6* and *E7* genes in HeLa cells before and after treatment with *Lactobacillus* crispatus and *Lactobacillus* rhamnosus culture supernatants.

**Results:**

The expression of *CASP3* and autophagy genes in HeLa cells was de- creased after treatment with lactobacilli culture supernatants. However, this de- crease was not significant for *PRKAA2* when compared with controls. In addition, expression of HPV *E6* was significantly decreased after treatment with lactobacilli culture supernatants.

**Conclusion:**

Lactobacilli culture supernatants can decrease expression of *ATG14* and *BECN1* as well as the HPV *E6* oncogene. It has been demonstrated that the main changes occurring during cervical carcinogenesis in cell machinery can be reversed by suppression of HPV oncogenes. Therefore, downregulation of HPV *E6* by lacto- bacilli may have therapeutic potential for cervical cancer. As the role of autophagy in cancer is complicated, further work is required to clarify the link between downregula- tion of autophagy genes and antiproliferative effects exerted by lactobacilli.

## Introduction

Lactobacilli are a group of beneficial microorganisms (probiotics) with beneficial effects for the host when administered in sufficient amounts (1). They are the normal flora of vagina and have been shown to have protective effects against sexually transmitted viral diseases. In addition, lactobacilli found in the gastrointestinal tract have gained remarkable attention because of their health promoting characteristics (2). The lactobacilli supernatant has been demonstrated to have components with neutralizing activity against human immunodeficiency virus (HIV) and herpes simplex virus (HSV) (3). *Lactobacillus* crispatus (*L.crispatus*) and *Lactobacillus* rhamnosus (*L. rhamnosus*) are among the most frequently occurring species in vagina of healthy women (2). Previous studies have indicated that *L. crispatus* but not *L. rhamnosus* has protective effects against bacterial vaginosis. Furthermore, *L. crispatus* SJ-3C-US has been shown to have the most potent anti-cancer effects among lactobacilli (2,4). Common vaginal lactobacilli have been shown to exert cytotoxic effects on cervical tumor cells but not on normal cells, in a manner which is independent of pH and lactate concentration. However, apoptosis has also been shown to be inhibited by lactobacilli supernatants (4). 

Autophagy is a conserved process which controls cell fate along with apoptosis (5). Autophagy is a catabolic pathway illustrated by the construction of double-membrane vesicles, namely autophagosomes. These structures surround cytoplasmic organelles and proteins, and then fuse with lysosomes which degrade their content (6). Since during the process of autophagy unnecessary or dysfunctional cellular components are degraded, autophagy has been known as a mechanism that promotes cellular survival during starvation by maintaining cellular energy (7). Several genes have been implicated in the process of autophagy. An important step in autophagy is phosphorylation of phosphatidylinositol (PtdIns) by a PtdIns 3-kinase. *ATG14* is a specific subunit of one of the PtdIns 3-kinase complexes which targets the complex to the site of autophagosome formation, thus arranging the complex to take part in autophagy (8). *BECN1* is a coiled-coil protein which directly interacts with the anti-apoptotic B-cell lymphoma-2 (Bcl-2) protein (9). It is therefore recognized as an important regulator of autophagy and shown to control the autophagic process by regulating PtdIns3KC3-dependent generation of PtdIns 3-phosphate and the subsequent recruitment of other autophagy related proteins (10). Evidence regarding the role of *BECN1* in autophagy came from at least 2 independent experiments. First, autophagy was impaired in *BECN1* +/− mice (11) and second, *BECN1* was downregulated in human breast MCF7 carcinoma cells (12). AMP activated protein kinase (AMPK) is a key energy sensor which controls cellular metabolism to keep energy homeostasis and shown to promote autophagy through direct phosphorylation of Ulk1 (13). In this study, we aimed to analyze the expression of these autophagy related genes [*ATG14*, *BECN1* and alpha 2 catalytic subunit of AMPK (*PRKAA2*)] and an effector caspase (s) in addition to human papilloma virus (HPV) *E6* and *E7* oncogenes in the HeLa cervical cancer cell line after treatment with *L. crispatus* and *L. rhamnosus* culture supernatants. *L. crispatus* unlike *L. rhamnosus* is not available in the form of commercial probiotic microcapsules. Considering the mentioned health promoting effects of *L. crispatus*, it is reasonable to compare the effects of these two lactobacilli on the expression of mentioned genes to assess the potential of *L.crispatus* as a probiotic. 

The most distinguished risk factor for cervical cancer is HPV infection. Cervical cancer incidence does not parallel the high prevalence of HPV infection (14). The majority of HPV infections and infectioninduced lesions are temporary or intermittent and resolved spontaneously (15). Therefore HPV infection alone is inadequate, and other environmental and host factors such as cervical microbial flora and infections may participate in the process of carcinogenesis (4). 

HeLa is a cervical carcinoma cell line that contains HPV18 DNA. It has been demonstrated that HeLa cells as well as most cervical carcinomas have wildtype p53 and p105^Rb^ genes. HPV E6 and E7 proteins, which are expressed by most cervical carcinomas, have been shown to counteract with cellular tumor suppressor function and mask the growth inhibition machinery in these cells (16). 

## Materials and Methods

### Cell culture

This study was approved by the Ethical Committee of Tehran University of Medical Sciences. The human cervical cancer (HeLa) cell line was obtained from Pasteur Institute, National Cell Bank of Iran. Cells were cultured in RPMI 1640 medium containing 10 % heat inactivated fetal calf serum (Invitrogen, USA), 603 1.5% HEPES (Invitrogen, Carlsbad, CA, USA) and 1% penicillin/streptomycin (Invitrogen, USA). Cells were maintained as monolayer cultures at 37˚C in a humidified 5 % CO_2_ atmosphere, and were then plated 24 hours before treatment to allow adhesion.

### Preparation of supernatants from Lactobacillus cultures

*L. crispatus* strain SJ-3C-US (LbC) and *L. rhamnosus* strain GG (LbR) were grown in de Man Rogosa Sharpe (MRS) broth (Merck, pH=6.5) at 37˚C for 24 hours under microaerophilic conditions. Overnight bacterial cultures with a concentration of 2*10^9^ c.f.u./ ml were centrifuged at 1100 ×g for 15 minutes at 4˚C. To eliminate the remaining bacteria and debris, the lactobacilli supernatants (LS) were filtered through a 0.2 mm membrane filter. The pH of the MRS broth was 6.5; however, in the preparation of LS we decreased the pH to 4 ± 0.1. To examine whether lactate produced by the two lactobacilli and pH change would affect the experiments, the lactate concentration in LS was checked using a Lactate Randox kit (Randox Laboratories). Non-cultured MRS broth adjusted to pH=4 with lactate was used in co-culture experiments. The following were tested: *L. crispatus* supernatant (LCS), pH=4; *L. rhamnosus* supernatant (LRS), pH=4; MRS, pH=6.5; MRS, pH=4; adjusted with lactate (MRS with Lactate: MRL). 

### 3-(4,5-dimethylthiazol-2-yl)-2,5-diphenyltetrazolium bromide (MTT) assay

Cell growth inhibition was measured by a 3-(4,5-dimethylthiazol-2-yl)-2,5-diphenyltetrazolium bromide (MTT) assay kit (Sigma, St. Louis, MO). A total of 10^4^ cells were seeded in each well containing 100 ml standard medium. After overnight growth, cells were treated for 24 hours with 1, 2, 5, 10, 15, 20, 40, 60, 80 and 100% (v/v) lactobacilli culture supernatants. Plates were then incubated at 37˚C under 5% (v/v) CO_2_. Cell viability was determined according to: 

Viability (percentage of control)=[(absorbance sample-absorbance blank)/[absorbance controlabsorbance blank)]×100. 

RNA isolation, cDNA synthesis and quantitative real time-polymerase chain reaction (qRT-PCR). 

The FastPure RNA kit (Takara Bio, Ohtsu, Japan) was used to isolate total RNA from cultured cells. RNA concentration was assessed using a Nanodrop 2000c spectrophotometer (Thermo Scientific). Changes in mRNA expression were analyzed by quantitative PCR (qPCR) after reverse transcribing 1 μg RNA from each sample with the PrimeScript RT reagent kit (Takara Bio, Ohtsu, Japan). qRT-PCR was undertaken on a Rotor-Gene 3000 (Corbette Research, Australia) using SYBR Premix Ex Taq (Takara Bio, Ohtsu, Japan). The sequences of primers used are given in table 1. Thermal cycling conditions were an initial denaturation at 95˚C for 1 minute, and 40 cycles of 95˚C for 15 seconds and 65˚C for 1 minute. The PCR was performed in a final volume of 20 µl containing 10 µl SYBR Green master mix, 2 µl cDNA, 0.5 µl each forward and reverse primer (10 pmol) and 7 µl nuclease-free water. Experiments were performed in duplicate for each data point. Glyceraldehyde 3-phosphate dehydrogenase (GAPDH) mRNA was amplified as a normalizer, and fold changes in each target mRNA expression relative to GAPDH were calculated. Melting curve analysis was used to validate whether primers yielded a single PCR product. 

### Statistical analysis

The Relative Expression Software Tool-RG©version 3 (Qiagen, Korea) was used for comparison of the total expression ratio of mentioned genes between treated and control cells. 

SPSSv.15.0.1 (SPSS Inc., Chicago, IL) was used to apply Mann-Whitney test for comparison of inhibitory concentration 50% (IC_50_)(concentration giving half-maximal inhibition) of cells treated with LS with pHand lactate adjusted and pretreated controls. All data were expressed as mean ± standard error (SE) of three separate MTT experiments. P<0.05 was considered as statistically significant. 

## Results

### Supernatant of *L. crispatus* and *L. rhamnosus* inhibit HeLa cell proliferation

**Fig.1:**
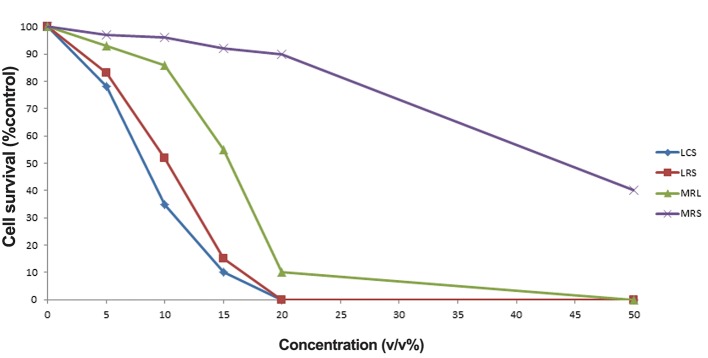
Cell growth inhibitory effects of different concentrations of LCS, LRS, MRS and MRL on HeLa cells. LCS; *Lactobacillus* crispatus supernatant, LRS; *Lactobacillus rhamnosus* supernatant, MRS; de Man Rogosa Sharpe and MRL; MRS with lactate.

The IC_50_ value of LCS and LRS against HeLa cells was 10% (v/v). The cytotoxic effects of LCS and LRS against HeLa cells were higher than those of MRS and MRL (MRS with pH adjusted to that of LCS and LRS) (P<0.05, Fig .1). Cytotoxicity effect of LCS was significantly higher than LRS (P<0.01).

**Table 1:** Primer sequences


Primer	Sequence	Productsize(bp)

*GAPDH*	F:CCTGGCGTCGTGATTAGTGAT	131
*GAPDH*	R:AGACGTTCAGTCCTGTCCATAA	
*ATG14*	F:GCGCCAAATGCGTTCAGAG	162
*ATG14*	R:AGTCGGCTTAACCTTTCCTTCT	
*BECN1*	F:ACCTCAGCCGAAGACTGAAG	94
*BECN1*	R:AACAGCGTTTGTAGTTCTGACA	
*PRKAA2*	F:AGGTGATCAGCACTCCAAC	125
*PRKAA2*	R:AAATCGGCTATCTTGGCATTCA	
HPV18 *E6*	F:CGCGCTTTGAGGATCCAA	195
HPV18 *E6*	R:TATGGCATGCAGCATGGG	
HPV18 *E7*	F:AACATTTACCAGCCCGACGA	106
HPV18 *E7*	R:TCGTCTGCTGAGCTTTCTAC	


### L. crispatus and L. rhamnosus downregulate CASP3 and two autophagy genes

**Fig.2:**
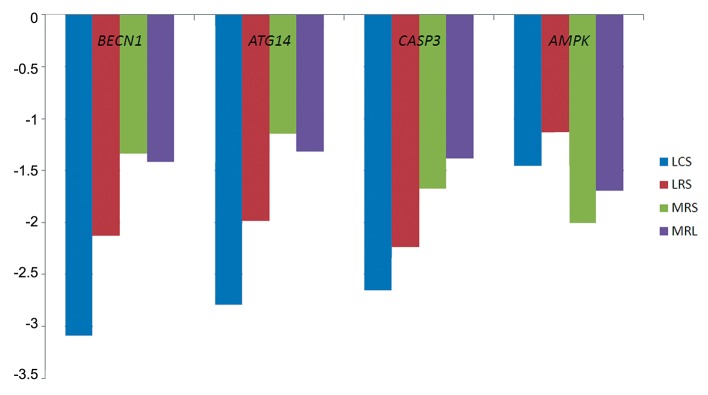
The effects of LCS, LRS, MRS and MRL on the expression of *CASP3*, *ATG14* *BECN1* and AMPK (*PRKAA2*). LCS; *Lactobacillus* crispatus supernatant, LRS; *Lactobacillus rhamnosus* supernatant, MRS; de Man Rogosa Sharpe and MRL; MRS with lactate.

After 4 hours treatment of HeLa cells with 10% (v/v) LS, LCS and LRS downregulated mRNA levels of all genes (P<0.001) except for PRKKA2 (P=0.47) when compared with MRS and MRL (Fig .2). 

### L. crispatus and L. rhamnosus downregulate HPV18 E6 oncogene

**Fig.3:**
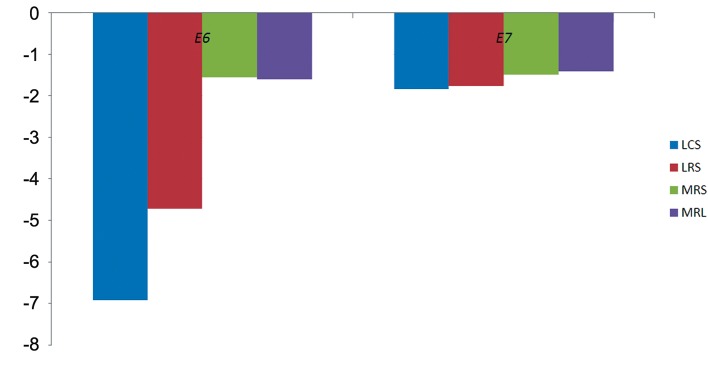
The effects of LCS, LRS, MRS and MRL on the expression of HPV *E6* and *E7* oncogenes. LCS; *Lactobacillus* crispatus supernatant, LRS; *Lactobacillus* rhamnosus supernatant, MRS; de Man Rogosa Sharpe and MRL; MRS with lactate.

After 4 hours treatment of HeLa cells with 10% (v/v) LS, LCS and LRS downregulated *E6* by 6.93and 4.74-fold respectively (P=0.001). However, *E7* was not significantly downregulated when compared to MRS and MRL (P=0.6, Fig .3). 

## Discussion

Apoptosis and autophagy are two important processes in defining cell fate. The functional link between these two processes has been shown to be complex. In some situations, autophagy comprises a stress adaptation to evade cell death (and thus suppresses apoptosis), while in other circumstances, it is an alternative cell-death pathway (17). Although autophagy is generally believed to be a tumor-suppressive process, the link between autophagy and cancer is not straightforward. It seems that different steps of autophagy have different functions in tumorigenesis and tumor survival (18). For instance, based on a mouse model of lymphoma, it has been demonstrated that inhibition of autophagy with either chloroquine or *ATG5* short hairpin RNA (shRNA) can increase p53 induced apoptosis or enhance tumor regression in response to alkylating drug therapy. Consequently, it has been suggested that autophagy inhibitors such as chloroquine can be used in combination with apoptosis inducing agents in human cancers (19). In the current study, we showed that that the main cause of HeLa cell death by LS was not the level of acidity. It could therefore be the result of a substance other than lactate in LS that causes cervical tumor cell death. In addition, we demonstrate by the means of qRT-PCR that LS can downregulate expression of two genes with critical roles in autophagy as well as *CASP3*. *CASP3* downregulation by LS has been reported previously (4). However, this is the first report regarding downregulation of autophagy genes by lactobacilli. As autophagy can have both tumor suppression and pro-survival effects in cancer, further work is required to elucidate the link between downregulation of autophagy genes and antiproliferative effects exerted by lactobacilli. 

Previously, it has been demonstrated that a probiotic lactic acid bacterium named *bifidobacterium adolescentis* SPM1005-A has antiviral activity in the SiHa cervical cancer cell line through expression suppression of *E6* and *E7* oncogenes. Although this was observed for both at the transcript level, the protein level of only *E6* protein decreased when compared with that in the control (20). Here we show that the transcript level of only *E6* decreases significantly after treatment with lactobacilli supernatants. To the best of our knowledge, this is the first report showing downregulation of the HPV *E6* oncogene by lactobacilli. Since suppression of endogenous HPV oncogenes can reverse cervical carcinogenesis (16), antiproliferartive effects of these two probiotics may thus be exerted, at least partly, via downregulation of the HPV18 *E6* oncogene. 

## Conclusion

*L. crispatus* and *L. rhamnosus* have antiproliferative effects on HeLa cervical cancer cells. The mechanism of this effect is not clear. However, this cytotoxicity can be exerted at least partially via downregulation of HPV oncogenes. As therapeutic effects of lactobacilli in cancers are currently being evaluated, the results of this study provide further evidence in this regard. 
